# Chemical Alarm Cues Are Conserved within the Coral Reef Fish Family Pomacentridae

**DOI:** 10.1371/journal.pone.0047428

**Published:** 2012-10-18

**Authors:** Matthew D. Mitchell, Peter F. Cowman, Mark I. McCormick

**Affiliations:** ARC Centre of Excellence for Coral Reef Studies and School of Marine and Tropical Biology, James Cook University, Townsville, Queensland, Australia; King Abdullah University of Science and Technology, Saudi Arabia

## Abstract

Fishes are known to use chemical alarm cues from both conspecifics and heterospecifics to assess local predation risks and enhance predator detection. Yet it is unknown how recognition of heterospecific cues arises for coral reef fishes. Here, we test if naïve juvenile fish have an innate recognition of heterospecific alarm cues. We also examine if there is a relationship between the intensity of the antipredator response to these cues and the degree to which species are related to each other. Naïve juvenile anemone fish, *Amphiprion percula*, were tested to see if they displayed antipredator responses to chemical alarm cues from four closely related heterospecific species (family Pomacentridae), a distantly related sympatric species (*Asterropteryx semipunctatus*) and a saltwater (control). Juveniles displayed significant reductions in foraging rate when exposed to all four confamilial heterospecific species but they did not respond to the distantly related sympatric species or the saltwater control. There was also a strong relationship between the intensity of the antipredator response and the extent to which species were related, with responses weakening as species became more distantly related. These findings demonstrate that chemical alarm cues are conserved within the pomacentrid family, providing juveniles with an innate recognition of heterospecific alarm cues as predicted by the phylogenetic relatedness hypothesis.

## Introduction

Accurate assessment of predation risk is vital to the success of any individual, as early detection of a predator enhances the chances of prey survival [1], [2]. However, to be successful antipredator defences must be balanced with other fitness enhancing behaviours (e.g. feeding and reproduction) [1]. This leads to a selective pressure on individuals to acquire information about current predation risks within their environment, in order to modify their antipredator behaviour to reflect their current level of risk. Such a strategy should optimise the trade-off between predator avoidance and other fitness enhancing behaviours [3], [4]. Individuals that are also able to detect and respond to alarm cues from heterospecific species that share a common predator will also have a fitness advantage [5], [6]. The use of heterospecific alarm signals in risk assessment is common across multiple taxa: birds [7], mammals [8], freshwater fishes [9], amphibians [10], [11], insects [12] and crustaceans [13]. Furthermore, information from heterospecific individuals may be more valuable than that from conspecifics, as heterospecific species may impose a lower competitive cost than a conspecific [14].

In aquatic systems, chemical cues along with visual cues are the primary sources of information for assessment of predation risk [15]. Released from specialised cells in the epidermis, following mechanical damage during a predation event, chemical alarm cues provide early warning of potential danger for other individuals within the local area [16], enhancing chances of survival [9], [17], [18]. The importance of chemical cues is highlighted by the simultaneous evolution of chemical alarm cues within most aquatic taxa found in both freshwater and marine environments (reviewed in [19]). They are of particular importance in complex or turbid habitats where visual cues are reduced [15], [20]. Unsurprisingly, prey also use chemical alarm cues derived from heterospecifics to gain information about local predation risks [9], [10], [21].

Responses to heterospecific alarm cues may arise from one of two non-exclusive mechanisms: 1) Individuals may possess an innate recognition of alarm cues common to closely related species (the “phylogenetic relatedness hypothesis”) [10], [11], [22]; or 2) Individuals may acquire recognition of relevant alarm cues through experience (the “ecological coexistence hypothesis”) [6], [22], [23]. The phylogenetic relatedness hypothesis proposes that alarm cues are conserved within taxonomic groups and thus individuals are able to generalise the recognition of their own alarm cue to those of closely related heterospecific species, as the composition of both alarm cues should be similar having been derived from a recent common ancestor [10], [11]. Individuals should therefore display a stronger antipredator response to closely related species and a weaker response to species that are more distantly related, irrespective of whether the species are allopatric or sympatric [11], [24], [25]. Strong evidence supporting the phylogenetic conservation of alarm cues is provided for grey tree frog tadpoles, *Hyla versicolor* [11] and Ostariophysan fishes [26].

In contrast, the ecological coexistence hypothesis suggests that responses to alarm cues from heterospecific species arise due to individuals co-existing with species that are part of the same prey guild [11], [23]. As both species share a common predator it is beneficial to respond to each other’s alarm cues as it will enhance early detection of a predator. Such responses may arise through learning as individuals gain experience with the predator-prey community in their local environment [21], [27], or they may be innately fixed through co-habitation with sympatric prey guild members over several generations [11]. Support for this hypothesis is often confounded by the use of wild caught individuals, as it is not possible to control for experience prior to collection. Consequently, it is not possible to make definitive conclusions about how observed responses to heterospecific alarm cues arose. Interestingly, two studies suggest that ecological coexistence may play an important role in modifying responses to phylogenetically conserved responses to heterospecific alarm cues [6], [23].

For fishes, how responses to heterospecific cues arise is still open to debate. The ability to acquire learnt recognition of heterospecific cues has been demonstrated across a wide range of fish taxa: minnows [28], sticklebacks [9], cichlids [29], gobies [30] and salmonids [24]. This suggests that ecological coexistence plays a significant role in acquiring recognition of heterospecifics at the individual level. However, support for the conservation of chemical alarm cues within taxonomic groups varies greatly. Of the taxonomic groups tested to date, alarm cues appear to be highly conserved within the superorder Ostariophysan (where the putative chemical alarm cue hypoxanthine-3-N-oxide has been identified [26]) and the salmonid family [24]. Other studies on wild darters, genus *Etheostoma* [31]–[33] and two species of coral reef gobies *Asterropteryx semipunctatus* and *Brachygobius sabanus* [30] provide inconclusive support for either hypothesis. Indeed, a more rigorous empirical assessment is still needed to address the phylogenetic relatedness hypothesis among fishes and other vertebrates, and the extent to which phylogeny determines the magnitude of antipredator responses. The answers to these questions are particularly important in understanding antipredator behaviour in species-rich habitats such as coral reefs.

Recent studies have highlighted the importance of chemical alarm cues in predator-prey dynamics for coral reef fishes, particularly for newly settled recruits [18], [34]. Recruits are exposed to a period of extremely high predation following settlement [35] and must rapidly learn to recognise predators to survive. During this period, chemical alarm cues play a crucial role in predator recognition [34], [36] and survival [18]. Given that many species recruit to reefs around the new moon period and are likely exposed to a similar suit of predators, the ability to access information from heterospecifics will facilitate the rapid acquisition of predator identities and increase an individual’s chances of surviving, particularly if they have an innate recognition of alarm cues from heterospecific species that share a common predator. However, to date only a goby, *Asterryoptryx semipunctatus* has been shown to be able to display antipredator responses to heterospecific alarm cues.

The aim of this study was firstly, to see if a common coral reef fish had an innate knowledge of heterospecific alarm cues at the time of settlement and secondly, to assess whether there was a relationship between the intensity of response to heterospecific cues and the extent to which they were related to each other, indicative of a phylogenetically conserved alarm cue. To do this we tested naïve juvenile anemone fish, *Amphiprion percula* (family: Pomacentridae), for an innate antipredator response to a range of chemical alarm cues from four heterospecific species within the pomacentrid family. They were also tested for their response to an alarm cue from a distantly related prey guild member, *Asterropteryx semipunctatus*, and a saltwater control. We then compared the intensity of the response to the heterospecific alarm cues to the time of divergence from the nearest common ancestor shared between *A. percula* and each of the heterospecific species.

## Methods

### Ethics Statement

Research was carried out under approval of the James Cook University animal ethics committee (permit: A1067) and according to the University’s animal ethics guidelines. Fish collections around Orpheus Island, Great Barrier Reef were carried with permission of the Great Barrier Reef Parks Authority (permit: G10/33239.1) and Queensland Government Department of Primary Industry and Fisheries (permit: 103256).

### Study Species

*Amphiprion percula* is a member of the highly diverse and abundant Pomacentridae family that inhabit coral reefs throughout the tropics. While it is found in the same general habitat to the heterospecific species in this study they display distinct micro-habitat difference due to its symbiotic relationship with certain anemone species [37]–[41]. Consequently the extent to which they are exposed to their alarm cues should be similar for all species. Additionally, all species in this study are targeted by similar range of predators [42]–[44]. The heterospecific species were chosen based on their phylogenetic relationship to *A. percula*; *Amphiprion melanopus* is a closely related congeneric species, *Pomacentrus moluccensis* and *Acanthochromis polyacanthus* are both from different genera with the Pomacentrinae sub-family, *Chromis atripectoralis* is from the Chrominae sub-family, one of the most basal groups with the Pomacentridae [45] and *Asterropteryx semipunctatus* are from the distantly related Gobiidae family. All species are known to possess chemical alarm cues ([20], [30], [36], Mitchell unpublished data).

### Collection and Maintenance

*A. percula* juveniles were captive bred and reared to settlement at the James Cook University aquarium facility, following the methods outlined in Dixon et al. [46]. Juveniles were maintained in three 40-l flow-through aquaria and fed 2/4 NRD marine food pellets (Spectrum Aquaculture) until they reached ∼20 mm in length, at which point they were large enough to use in the experiments. Captive breeding ensured that the fish would be completely naïve to the alarm cues of other species.

The five donor species were either taken from captive breed stocks or collected from the wild. *A. melanopus* and *Ac. polyacanthus* were captive bred at the university aquarium facility and reared to the same size as *A. percula*. All other species (*P. moluccensis*, *C. atripectoralis* and *As. semipunctatus*) were collected from coral reefs around Orpheus Island, Great Barrier Reef, Australia. Juveniles of each species were collected using hand nets and anaesthetic clove oil. All fishes were maintained in separate 40-l flow-through aquaria and fed *ad libitum* twice a day with 2/4 NRD marine food pellets (Spectrum Aquaculture).

### Stimulus Preparation

Alarm cues were prepared fresh directly before being used in each trial. One individual per treatment was sacrificed by a quick blow to the head and placed in a disposable Petri dish. Using a clean scalpel blade, 15 superficial cuts were made along each flank of the fish. Fish were rinsed with 15-ml seawater and the solution was filtered through filter paper to remove any solid material.

### Observation Tanks

Conditioning and recognition trials were conducted in 11-l flow-through aquaria (30×20×15 cm). Each tank had a 2-cm layer of gravel, a small terracotta pot (5-cm diameter) for shelter at one end and an air stone at the opposite end. An injection tube was attached to the air stone tube to allow food and odours to be introduced with minimal disturbance to the fish. A 3×6 grid (4×5 cm) was drawn onto the front of each tank. Each tank was surrounded on three sides with black plastic to visually isolate the fish and a black plastic curtain was hung in front of the tanks to create an observation blind.

### Recognition Trials

Individual *A. percula* were placed into test aquaria and left to acclimate for two days. On the morning of testing fish were fed 30-ml *Artemia* solution (containing ∼200 individuals per ml) and left for at least 1 h before testing began. Trials were conducted between 0800 h and 1600 h each day. Prior to the start of trials the flow-through system was turned off and 10-ml of seawater were withdrawn and discarded from the tube, to remove any stagnant water, and a further 20 ml were withdrawn and retained for flushing. Trials consisted of an initial 2-min feeding period, a 5-min pre-stimulus observation period and a 5 min post-stimulus period. At the start of the 2-min feeding period 30-ml of *Artemia* were injected into the tank followed by 10-ml seawater to flush the tubing to allow feeding rates to stabilise. Once feeding rates had stabilised the 5-min pre-stimulus observation commenced. At the end of the observation period 15-ml of stimulus odour were injected followed by 10-ml of seawater for flushing and the post-stimulus observation period began 1 min later. The stimuli consisted of one of the five skin extracts or a saltwater control. Stimuli were assigned randomly to the tanks. Individuals were tested for their response to one skin extract only. A total of 150 fish were tested (18–20 individuals per treatment).

The behaviour of the focal fish was observed during the pre- and post-stimulus observation periods. We quantified two response variables: foraging rate and distance from shelter. Decreased foraging rate and distance from shelter are well known antipredator responses displayed by a number of prey species, including coral reef fishes [19], [36], [47]. Foraging rate included all feeding strikes irrespective of whether they were successful at capturing prey. For distance from shelter, the horizontal and vertical locations of the fish in the tank were recorded every 15s, using the grid drawn on the side of the tank. The position of the fish in the tank was then converted into a linear distance from shelter using the dimensions of the grid squares and Pythagoras’s theorem.

### Identification of Phylogenetic Relatedness

**Figure 1:**
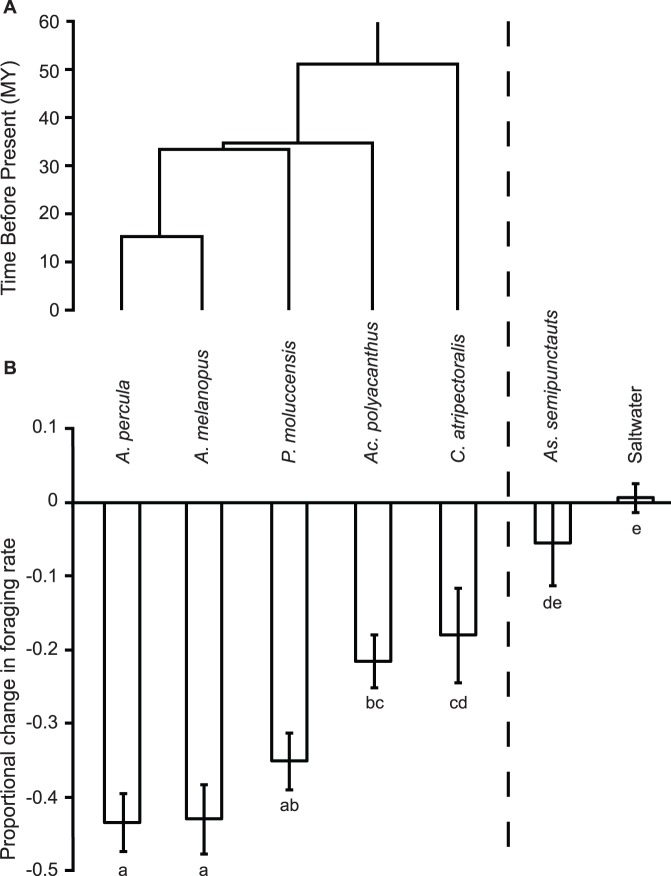
The phylogeny of Pomacentridae study species and antipredator response to heterospecific alarm cues. The phylogenetic relationship and antipredator response of *Amphiprion percula*, to heterospecific family members (*Amphiprion melanopus* ¸ *Pomacentrus moluccensis*, *Acanthochromis polyacanthus* and *Chromis atripectoralis*), a distantly related sympatric prey guild member (*Asterropteryx semipunctatus*) or a saltwater control. a) A chronogram (modified from [45]) displaying the divergence times of the MRCA of the focal species, *A. percula* to each of the heterospecific donor lineages within the family Pomacentridae. Ages are calibrated to millions of years before present. b) The mean change in foraging rate (±SE) of juvenile *A. percula* exposed to the chemical alarm cues of five heterospecific species and a saltwater control. Fishes are ordered with respect to their relatedness to *A. percula*. Letters below bars indicate Tukey’s groupings.

To assess if the magnitude of an antipredator response to a heterospecific alarm cue is regulated by the phylogenetic relatedness of the focal species to the heterospecific species, we used the ‘time of divergence’ of our focal species (*A. percula*) and the most recent common ancestor (MRCA) to the heterospecific lineage in question. We made use of a recently published chronogram (time-calibrated phylogeny) of the family Pomacentridae [45] to find the divergence time of the MRCA of *A. percula* and the heterospecific alarm cue donors (Table 1). The pomacentrid chronogram was reconstructed using Bayesian age estimation methods and fossil calibration techniques (see methods [45]). It includes all of the pomacentrid taxa used in this study and all major lineages of the family Pomacentridae. The timing of divergence (T_D_) of each pomacentrid heterospecific from *A. percula* was taken as the age of the MRCA of both lineages (T_MRCA_), minus the age of the node representing the origin of *A. percula* (T_Ap_; Fig. 1a; Table 1). This correction for the age of the *A. percula* lineage standardises the MRCA age to a metric that is specific to an ancestor node of *A. percula*.

**Table 1:** Divergence times of heterospecific lineages from *A. percula.*

Species	MRCA age (T_MRCA_)	MRCA distance (T_DP_)
	(MY)	(MY)
*A. percula*	2.7 (T_AP_)	0
*A. melanopus*	15.3	12.6
*P. moluccensis*	33.4	30.7
*Ac. polyacanthus*	34.7	32.0
*C. atripectoralis*	51.1	48.4

Ages are in millions of years (MY) before present and are taken from [45].

### Statistical Analysis

The proportional difference between pre- and post-stimulus behavioural observations were calculated and used as the raw data. The effects of test odour (the six fish alarm cues and saltwater) on foraging rate and distance from shelter of *A. percula* were analysed using individual 1-factor ANOVA’s. To account for ANOVA’s being run on two variables that were potential interrelated a Bonferroni adjustment was employed (adjusted α = 0.025). The ANOVAs revealed that only foraging rate was affected by test odour. The subsequent analyses were done on the foraging variable only. Tukey’s HSD post-hoc analysis was used to identify significant differences between responses to the test odours.

The relationship between the foraging response of individuals to the different pomacentrid chemical alarm cues and the divergence time between the different pomacentrid species and *A. percula* (T_D_) was investigated using a linear regression. Divergence time was used as the predictive variable and mean change in foraging rate was used as the response variable. For both analyses, the data was checked for outliers and residual analyses revealed that all data met the assumptions of homogeneity of variance and normality.

## Results

Test odour had a significant influence on *A. percula* foraging rate (*F*_ 6,111_ = 18.78, *p*<0.0001). Post-hoc tests showed that individuals displayed a significant reduction in foraging rate when exposed to alarm cues from conspecific *A. percula* and the heterospecifics *A. melanopus*, *P. moluccensis* and *Ac. polyacanthus* compared to the saltwater control and *As. semipunctatus* control (Fig. 1b). Individuals also showed a significant reduction foraging rate when exposed to *C. atripectoralis* compared to the saltwater control but not to the *As. semipunctatus* outgroup control. There was no difference in foraging rate between the saltwater control and *As. semipunctatus*, with feeding rate remaining constant throughout the trials (see Fig. S1 for mean pre- and post-exposure foraging rates). The 1-factor ANOVA on distance from shelter revealed there was no significant effect of test odour on *A. percula* (*F*_ 6,111_ = 1.38, *p* = 0.23).

**Figure 2:**
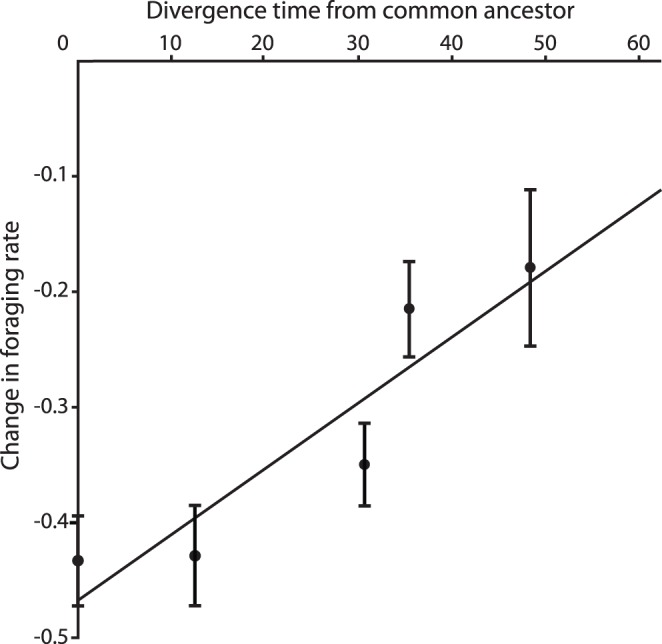
Relationship between antipredator responses and divergence times. The relationship between divergence time from the most recent common ancestor and the intensity of antipredator response of juvenile *Amphiprion percula* exposed to chemical alarm cues from various heterospecific species within the family Pomacentridae. Circles represent in the mean change in foraging rate (±SE) of *A. percula* to chemical alarm cues of each heterospecific species.

There was a significant relationship between the response to pomacentrid chemical alarm cues and the timing of divergence of the MRCA of the donor species and *A. percula*, which accounted for 66% of the intensity in antipredator response (*r* ^2^ = 0.66, *F*_1,88_ = 16.72, *p*<0.001; Fig. 2). The greatest reduction in foraging rate was displayed by individuals exposed to alarm cues from conspecifics and *A. melanopus*, the intensity of response then decreased as the donor species became more distantly related (Fig. 2).

## Discussion

Our results demonstrate that juvenile reef fish are able to detect and respond to heterospecific chemical alarm cues and that chemical alarm cues are conserved within the Pomacentridae family. Naïve juvenile *A. percula* displayed a significant reduction in foraging rate, when exposed to alarm cues from conspecific and heterospecific family members but not to alarm cues from the distantly related sympatric *As. semipunctatus* or the saltwater control. Additionally, the intensity of antipredator responses to heterospecific alarm cues diminished as the timing of divergence between the heterospecific cue and *A. percula* increased. These results support the findings of similar studies on salmonids [24] and invertebrates [10], [11], [23]. However, this is the first to demonstrate a strong relationship between phylogenetic relatedness and response intensity to heterospecific chemical alarm cues for a vertebrate species. This strong relationship suggests that the innate recognition of heterospecific cues by *A. percula* resulted from phylogenetic conservation of alarm cues as predicted by the ‘phylogenetic relatedness hypothesis’.

The ability to recognise and respond to heterospecific alarm cues will confer a significant survival advantage for reef fish throughout their lives but particularly during critical ontogenetic life history changes. Following an initial pelagic stage, larval reef fish recruit to reefs in pulses around the new moons throughout summer [48]. During this transition to the reef they enter an environment rich in generalist, opportunistic predators [49] and are subject to extremely high mortality (up to 60% in first 2 days [35]). Several studies have shown that coral reef fishes lack an innate antipredator responses to predator odours with regards to short term changes in risk perception [36], [47], [50], although Vail and McCormick [51] and Dixon et al. [46] suggest there maybe some level of innate recognition of certain predators. In the absence of innate predator recognition, there will be a strong selection pressure to rapidly gain information about potential predators, risky habitats or time periods in respect to predation. Consequently, individuals that are able to detect and respond to heterospecific alarm cues will increase their chances of detecting an active predator in their local vicinity and enhance their chances of surviving any subsequent attack.

The finding that *A. percula* responded to all the heterospecific alarm cues but not to *As. semipunctatus* (a prey guild member) demonstrates that alarm cues are conserved within the pomacentrid family. There was a strong relationship between the intensity of the antipredator response and the time since each heterospecific species diverged from its common ancestor with *A. percula*. These results support the predictions of the phylogenetic relatedness hypothesis, matching the findings of a number of previous studies on salmonids [24] and invertebrates [10], [11], [23]. In contrast, other studies investigating antipredator responses to heterospecific alarm cues found that responses to heterospecific cues were highly variable and there appeared to be no support for the phylogenetic relatedness hypothesis and only tentative support for the ecological coexistence hypothesis [13], [30], [52]. For example, while *As. semipunctatus* responded to both conspecific cues and heterospecific cues from *Gnatholepis anjerensis*, *G. anjerensis* responded to only conspecific cues [30]. Similarly, studies on freshwater darters [33] and sea urchins [52] found inconsistent patterns in responses to both conspecific and heterospecific cues. However, the previously mentioned studies were confounded by the fact that they used wild caught individuals rather than naïve individuals. Consequently, any innate responses to phylogenetically conserved alarm cues (if present) may have been modified through experience with coexisting prey guild members, masking any response patterns indicative of phylogenetically conserved cues.

While there is the potential that ecological coexistence could have influenced the innate patterns of response observed here, we would suggest it is unlikely that it caused the responses observed. The heterospecific species in this study were selected based on the consistency of overlap in habitat preference and exposure to common predators between *A. percula* and the heterospecific donor species. Given this, if ecological coexistence was causing the innate response to heterospecific alarm cues we would have expected the responses heterospecific cues to be uniform irrespective of the time of divergence from their common ancestor with *A. percula*. Additionally, we would have expected individuals to respond to *As. semipunctatus* as well. However, as we were unable to include any allopatric pomacentrid species there is the possibility that ecological coexistence might have influenced the responses observed. Dalesman et al. [23] and Dalesman and Rundle [6] demonstrated that ecological coexistence with heterospecific species can modify responses to phylogenetically conserved cues in snails, both at the population level, through coexistence over several generations, and at the individual level, through short term changes in prey guild community structure. Ecological coexistence may therefore play a secondary role in determining responses to phylogenetically conserved cues.

The capacity of any species to use heterospecific cues may depend on a number of intrinsic (e.g. the ability to detect heterospecific alarm cues) and extrinsic factors (e.g. how the individual interprets the relevance of the information once detected). The ability to detect heterospecific cues is dependent on them being sufficiently similar to the focal species’ own cues for recognition to occur. As demonstrated here, the intensity of response to heterospecific cues is directly related to the time of divergence from the most recent common ancestor. Species may not recognise heterospecific cues simply because the time since the two species diverged from their common ancestor was sufficient for the cues to become unrecognisable. Similarly, the rate at which such changes to the chemical cues occur will determine recognition patterns. For example, it is thought that chemical alarm cues play a significant role in immune system function for fishes [53], [54]. The composition of the alarm cues may therefore be affected in part by the need to maintain immune system functioning. Consequently, changes in the composition of alarm cues may be driven by changes in the environment (and immune challenges) to which the individual species is exposed. Such drivers may cause a rapid change in the chemical alarm cue of species that have moved into a markedly different ecological niche.

Extrinsic factors, such as the prey species’ ecology and life history, or the composition and foraging strategies of the predator community to which they are exposed may also influence how they respond to heterospecific alarm cues. The diversity of predatory species and their preferred foraging mode will likely influence responses of prey to heterospecific cues. Prey exposed to generalist predators (abundant on coral reefs), which target a broad range of species within a preys’ guild, will benefit from responding to heterospecific cues. Conversely, prey individuals exposed to specialist predators that target discrete types of species (or ontogenetic stages) within the prey guild may not gain benefits of responding to heterospecific cues, especially if the focal prey is rarely targeted by that predator [23]. Furthermore, life history strategies have the potential to strongly influence responses to heterospecific cues. Hazlett and McLay [13] suggested that the extent to which various crayfish responded to heterospecific cues did not depend on phylogenetic relatedness, but rather on whether they evolved in specious regions and had the ability to disperse widely. The dispersive pelagic larval phase of reef fish may help to maintain a prey fish’s responsiveness to heterospecifics, through the necessity for conservative risk assessment when settling to an environment that is highly patchy and unpredictable.

This study demonstrates that juvenile *A. percula* have an innate ability to recognise and respond to chemical alarm cues from closely related heterospecifics. The patterns of response strongly suggests that responses to heterospecific alarm cues result from a conserved chemical alarm cue within the Pomacentridae family as predicted by the phylogenetic relatedness hypothesis. Given the similarities between early life histories within reef fish, such baseline knowledge will enhance their capacity to detect risky situations and learn about the predators present in their new environment during a critical period in their life history. However, these innate patterns of response may not be permanently fixed. Previous studies have shown that responses to alarm cues can change throughout development, particularly in regards to how individuals perceive heterospecific cues [55]. As prey grow not only does perception of risk change with experience [56] but they will move into new prey guilds composed of different prey species and are exposed to different predators. Consequently, the patterns of responses to heterospecific cues will change throughout their lives to suit their current situation, incorporating new prey guild members and modifying innate responses as the perceived value of the information changes. To further understand the complexities of the predator-prey interactions that affect community composition and diversity on coral reefs, future studies need to look at how perception of risk alters with development and experience.

## Supporting Information

Figure S1 **The mean foraging rates (± S.E.) of juvenile** ***Amphiprion percula*** **before (shaded bars) and after (open bars) being exposed to the chemical alarm cues from conspecifics and five heterospecific species and a saltwater control.** A one-factor ANOVA revealed there was no significant difference in foraging rate between treatments foraging rates before being exposed to one of the odours (*F*_7, 140_ = 1.77, *p* = 0.097).
(PDF)
