# Supplementary material for: Chemical Alarm Cues Are Conserved within the Coral Reef Fish Family Pomacentridae
Source: PLoS One. 2012 Oct 18;7(10):e47428. doi: 10.1371/journal.pone.0047428 (PMC3475700; doi:10.1371/journal.pone.0047428)
Supplement: Figure S1 — The mean foraging rates (± S.E.) of juvenile Amphiprion percula before (shaded bars) and after (open bars) being exposed to the chemical alarm cues from conspecifics and five heterospecific species and a saltwater control. A one-factor ANOVA revealed there was no significant difference in foraging rate between treatments foraging rates before being exposed to one of the odours (F 7, 140 = 1.77, p = 0.097). (PDF) [file pone.0047428.s001.pdf]

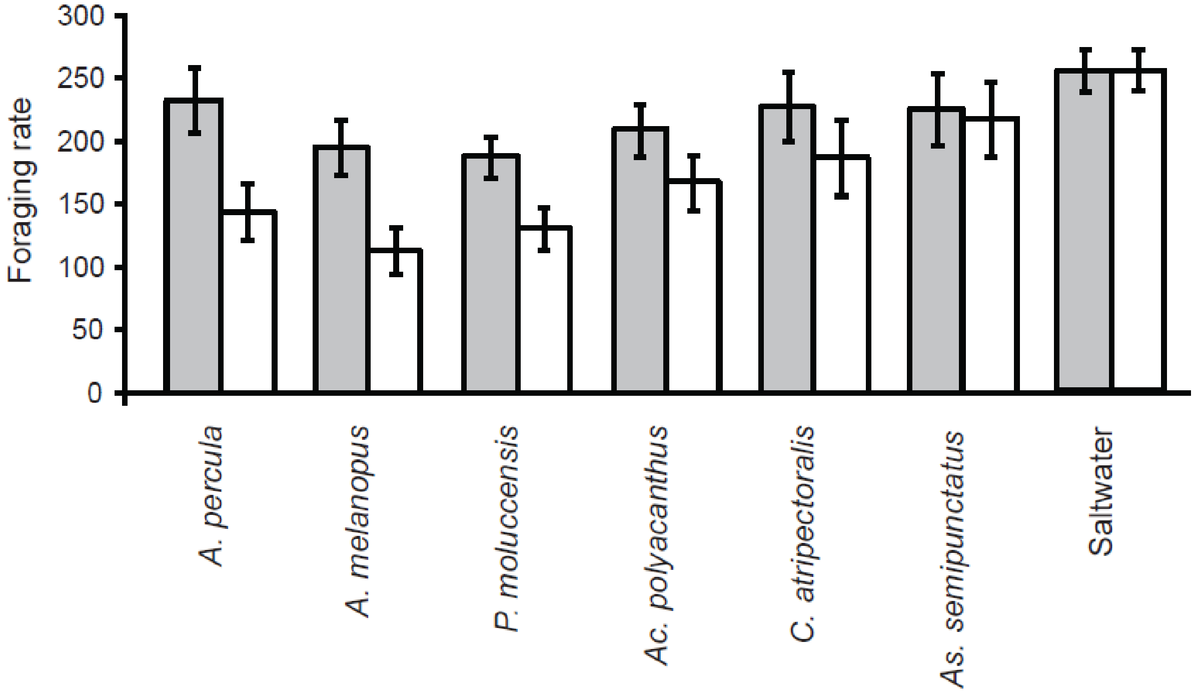

Figure S1. Mean foraging rate in pre- and post-stimulus trials. The mean foraging rates (± S.E.) of juvenile *Amphiprion percula* before (shaded bars) and after (open bars) being exposed to the chemical alarm cues from conspecifics, five heterospecific species and a saltwater control. A one-factor ANOVA revealed there was no significant difference in foraging rate between treatments foraging rates before being exposed to one of the odours ($F_{7, 140}$ =1.77, p= 0.097).
